# Can helmet decrease mortality of craniocerebral trauma patients in a motorcycle accident?: A propensity score matching

**DOI:** 10.1371/journal.pone.0227691

**Published:** 2020-01-13

**Authors:** Woo Sung Choi, Jin-Seong Cho, Yeon Sik Jang, Yong Su Lim, Hyuk Jun Yang, Jae-Hyug Woo

**Affiliations:** 1 Department of Emergency Medicine, Gachon University Gil Medical Center, Incheon, Republic of Korea; 2 Department of Emergency Medicine, Gil Medical Center, Gachon University College of Medicine, Incheon, Republic of Korea; Tsinghua University, CHINA

## Abstract

A helmet is critical for preventing head injuries during motorcycle accidents. However, South Korean motorcyclists have a lower prevalence of wearing a helmet, compared to developed countries. Therefore, we aimed to evaluate whether helmet wearing was associated with the clinical outcomes in Korean motorcycle accidents. Data were obtained from the Emergency Department-based Injury In-depth Surveillance database 2011–2015. We considered the patients had experienced a motorcycle accident and were only diagnosed with a craniocerebral trauma (CCT). The primary outcome was mortality and the secondary outcomes were the severity and hospitalization duration. The patients were separated whether they were wearing a helmet and the outcomes were compared using multivariate logistic regression after propensity score matching (PSM). Among 1,254,250 patients in the database, 2,549 patients were included. After PSM, 1,016 patients in each group were matched. The univariate analyses revealed that helmet wearing was associated with lesser severity (*P* < 0.001) and shorter hospitalization (*P* < 0.001). The regression analysis revealed that mortality was also lower in a helmet-wearing group (odds ratio: 0.34, 95% confidence interval: 0.21–0.56). In conclusion, wearing a helmet may reduce the mortality from a CCT after a motorcycle accident and associated with lesser severity and shorter hospitalization.

## Introduction

Between 2010 and 2014, approximately 415 South Koreans died each year because of motorcycle accidents. This mortality rate is less than the overall Korean car accidental death rate, which is approximately 2,595 people per year. However, motorcycle deaths account for 386 deaths per 10,000 accidents, compared to 175 accidental deaths per 10,000 car accidents [1]. Motorcycle-related deaths typically involve a craniocerebral trauma (CCT), and a helmet is the most important protective equipment for preventing or reducing the severity of CCTs [2][3]. Previous studies have examined the relationship between helmet wearing and mortality after motorcycle accidents. Some studies have reported that wearing a helmet was related to the low motorcycle-related mortality and other studies said that the legislation of helmet wearing for motorcyclists increased compliance and reduced the severity of accidents [4][5]. However, most previous studies evaluated mortality and severity independent of the affected body part, and it is difficult to accurately understand how wearing a helmet influences mortality and severity after a motorcycle-related CCT. Therefore, the present study aimed to evaluate whether wearing a helmet was associated with mortality, severity, and hospitalization duration among patients who only had a CCT diagnosis after a motorcycle accident.

## Methods

### Study design and subjects

This observational study evaluated patients with a motorcycle-related CCT who were entered into the Emergency Department-based Injury In-depth Surveillance (EDIIS) database between 2011 and 2015[6]. The database collects information from participating in emergency departments and is administered by the Korean Centers for Disease Control and Prevention (KCDC). The EDIIS database was launched in 2006 and was receiving data from 23 university hospitals by 2015. The present study evaluated data from 2011 to 2015.

**Fig 1:**
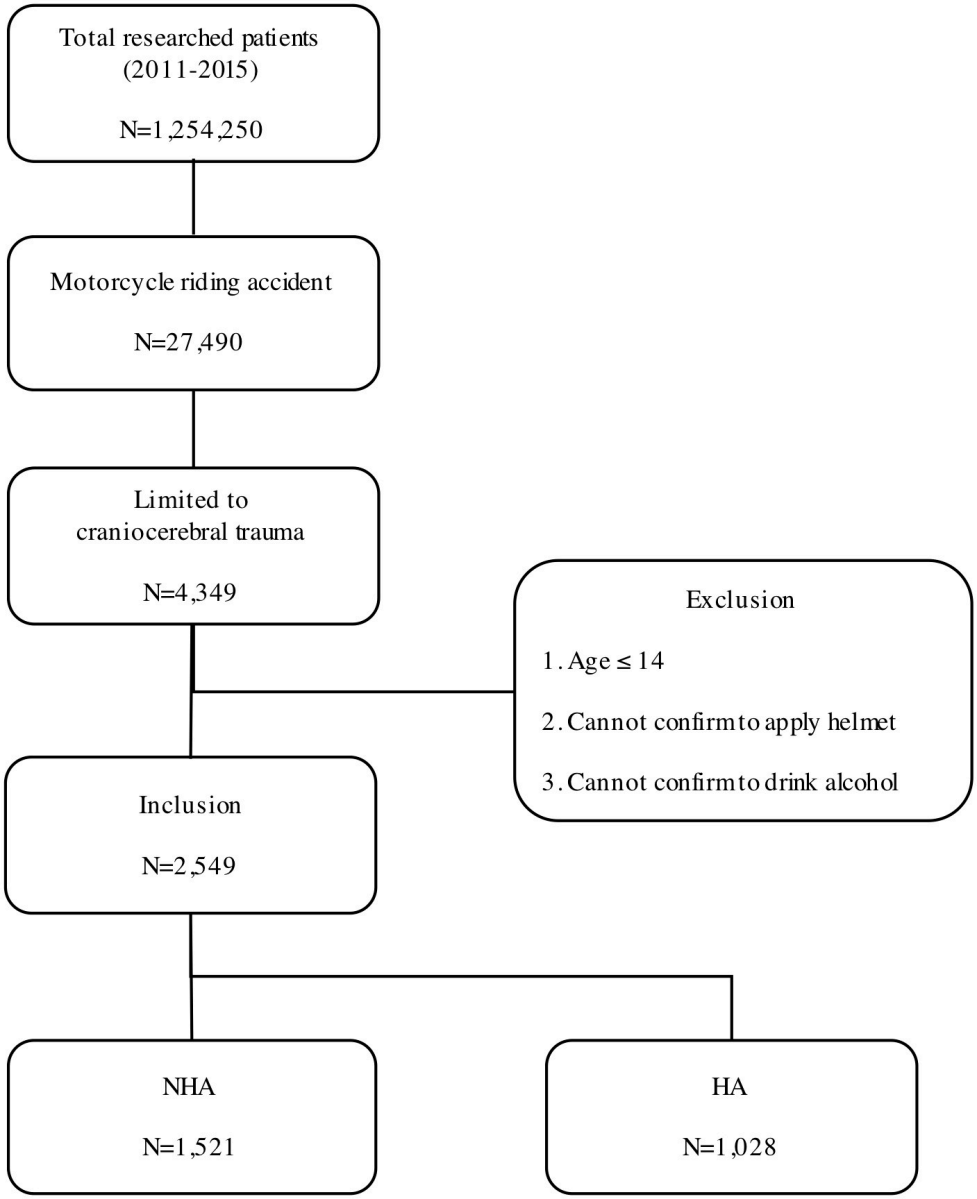
Flow diagram of the study population. NHA: Not worn a Helmet; HA: Worn a Helmet.

The study population included patients who had only a motorcycle accident as the ‘mechanism of injury’ and a CCT diagnosis, which was searched using the International Classification of Diseases-10 code (ICD-10) (Table in S1 Table). We excluded patients who were ≤14 years old, cases in which it was unclear whether the patient had been wearing a helmet, and cases in which it was unclear whether the patient had consumed alcohol (Fig 1). The criteria for the CCT diagnosis are listed in the EDIIS guidelines (Table in S1 Table). Based on the results, we compared the demographic characteristics and clinical outcomes of patients who had worn a helmet (the HA group) and patients who had not worn a helmet (the NHA group).

### Data collection

Twenty-three university hospitals have participated in the EDIIS project, and a coordinator collects data for each hospital. Prehospital data were gathered using prehospital medical records. Emergency room data were collected from the National Emergency Department Information System (NEDIS) and the patient’s medical records, admission records, and records from the patient’s admission to the intensive care unit and/or ward were collected from doctor and nurse’s medical record who were in charge of the patients.

### Ethical statement

This study’s design was reviewed and approved by the institutional review board of Gachon University Gil Hospital (GCIRB2016-242).

### Outcome measures

The primary outcome was defined as the relationship between mortality and wearing a helmet during the motorcycle accident. The secondary outcomes were defined as the relationship between wearing a helmet and the severity and/or hospitalization duration. Mortality was confirmed based on a record of death that was provided by the hospitals. Severity was calculated using the Excess Mortality Ratio-adjusted Injury Severity Score (EMR-ISS). The EMR-ISS is based on ICD-10 codes and is used to calculate the severity by summarizing the three highest squares of points (from 1 to 5) for various body parts (head, neck, torso, upper extremity, lower extremity, and non-traumatic injury [e.g., drowning]) [6][7]. Duration of hospitalization was defined as the time from the admission to the first instance of discharge, transfer, or death.

### Statistical analysis

Continuous variables were reported as mean and standard deviation or median and interquartile range (IQR). Student’s *t*-test was used to analyze normally distributed continuous variables and the Wilcoxon rank-sum test was used to analyze non-normally distributed continuous variables. Categorized variables were analyzed using the chi-square test. Propensity score matching (PSM) were used to evaluate the relationship between wearing a helmet and mortality. We used logistic regression to create a multivariate model to control for underlying confounders. Variables inserted in the full model were determined to be p <0.20 in univariate analysis, or to be considered clinically important by the authors. We performed multivariate logistic regression with backward elimination through likelihood-ratio tests and used the Hosmer-Lemeshow goodness-of-fit test to assess overall model performance of mortality. The PSM was used to adjust for differences in the HA and NHA groups’ demographic and general characteristics, such as age, sex, education, type of insurance, route of hospital admission, alcohol consumption, driver or passenger status, and time of the accident. Differences with a *P*-value of <0.05 were considered statistically significant, and all analyses were performed using STATA software (version 13.0).

## Results

During the 5-year study period, the EDIIS database contained records from 1,254,250 patients, although only 2,549 patients were considered eligible for our analyses. The 2,549 patients were categorized according to whether or not they had been wearing a helmet during the accident, and the two groups’ characteristics are shown in Table 1. The patients included a greater proportion of male patients, and male patients were significantly more common in the HA group, compared to the NHA group (*P* = 0.002). In addition, the NHA group was noticeably younger than the HA group (mean age: 39.2 years vs. 41.9 years). The NHA group also included a greater proportion of individuals who had consumed alcohol. The NHA group also included a greater proportion of passengers, compared to the HA group (13.3% vs. 3.7%). The NHA tended to be riding their motorcycle between midnight and 6:00 AM. The two groups exhibit similar education levels and types of insurance.

**Table 1:** Baseline characteristics and demographics feature of patients before PSM.

Characteristics	Total		NHA		HA		*P*-value
	N	41	N	%	N	%	
Total	2,549	100.0	1,521	100.0	1,028	100.0	
Gender							<0.001
Male	2,284	89.6	1,336	87.8	948	92.2	
Female	265	10.4	185	12.2	80	7.8	
Age (Years)							0.002
Mean ± SD	40.3±21.6		39.2±22.4		41.9±20.1		
Education	478		306		172		0.571
never go to school	25	5.2	13	4.2	12	7.0	
elementary school graduation	79	16.5	55	18.0	24	14.0	
middle school graduation	105	22.0	73	23.9	32	18.6	
high school graduation	148	31.0	87	28.4	61	35.5	
technical college graduation	5	1.1	3	1.0	2	1.2	
university graduation	39	8.2	26	8.5	13	7.6	
more than university	2	0.4	1	0.3	1	0.6	
unknown	75	15.7	48	15.7	27	14.0	
Insurance							0.099
NHI	861	33.8	535	35.2	326	31.7	
MAP	67	2.6	44	2.9	23	2.2	
Etc	1,619	63.5	940	61.8	679	66.1	
Unknown	2	0.1	2	0.1	0	0.0	
Method to come to hospital							0.037
Ground ambulance	1,959	76.9	1,204	79.2	755	73.4	
Air ambulance	8	0.3	6	0.4	2	0.2	
Etc	580	22.8	310	20.4	270	26.3	
Unknown	2	0.1	1	0.1	1	0.1	
Alcohol ingestion							<0.001
No	2,164	84.9	1,236	81,3	928	90.3	
Yes—Only driver	369	14.5	269	17,7	100	9.7	
Yes—All rider	16	0.6	16	1,1	0	0.0	
Role of patient							<0.001
Driver	2,309	90.6	1,319	86.7	990	96.3	
Passenger	240	9.4	202	13.3	38	3.7	
Time of accident (24hr)							<0.001
06–12	505	19.8	296	19.5	209	20.3	
12–18	790	31.0	444	29.2	346	33.7	
18–24	717	28.1	401	26.4	316	30.7	
00–06	537	21.1	380	25.0	157	15.3	

*Notes*: NHA: Not worn a Helmet; HA: Worn a Helmet; NHI: National Health Insurance; MAP: Medical Aid Program

The univariate analyses revealed differences between the two groups in most clinical variables (Table 2). For example, the NHA group exhibited lower values for systolic blood pressure (SBP), diastolic blood pressure (DBP), and Glasgow coma scale (GCS). The NHA group also exhibited higher values for heart rate (HR) and EMR-ISS. The NHA group exhibited a lower respiratory rate, although this difference was not significant. Finally, the NHA group exhibited a higher proportion of patients who died in the emergency room or who had prolonged hospitalization.

**Table 2:** Patient status and results of treatment before PSM.

Results	Total		NHA		HA		*P*-value
	N	%	N	%	N	%	
SBP (mmHg)	2,076		1,206		870		
Mean ± SD	132.4±27.1		131.0±28.5		134.4±25.0		0.005
DBP (mmHg)	2,076		1,206		870		
Mean ± SD	78.8±16.7		77.8±17.3		80.1±15.6		0.002
Heart rate	2,077		1,207		870		
Mean ± SD	83.0±17.8		83.8±19.2		81.9±15.5		0.017
Respiratory rate	2,075		1,206		869		
Mean ± SD	19.0±3.9		19.0±4.2		19.1±3.4		0.587
Glasgow coma scale	1,659		989		670		
Mean ± SD	14.1±2.7		13.9±2.9		14.4±2.3		<0.001
EMR-ISS	1,822		1,080		742		
Mean ± SD	18.7±15.8		20.2±17.2		16.5±13.1		<0.001
Results of ER treatment	2,549		1,521		1,028		<0.001
Discharge	1,324	51.9	720	47.3	604	58.8	
Transfer	275	10.8	181	11.9	94	9.1	
Admission	905	35.5	586	38.5	319	31.0	
Death	43	1.7	32	2.1	11	1.1	
Etc	2	0.1	2	0.1	0	0.0	
Results of admission	905		586		319		0.059
Discharge	635	70.2	402	68.6	233	73.0	
Transfer to other hospital	174	19.2	107	18.3	67	21.0	
Death	72	8.0	58	10.0	14	4.4	
Etc	24	2.7	19	3.2	5	1.6	
Admission date (days)	1,589		1,447		142		
Median (IQR)	13.0 (7.0–24.0)		14.0 (8.0–25.0)		6.0 (2.0–12.0)		<0.001
Death in hospital	2,549						<0.001
No	2,434	95.5	1,431	94.1	1,003	97.6	
Yes	115	4.5	90	5.9	25	2.4	

*Notes*: SBP: Systolic Blood Pressure; DBP: Diastolic Blood Pressure; EMR-ISS: Electrical Medical Records adjusted Injury Severity Score; ER: Emergency Room; SD: Standard Deviation; IQR: Interquartile Range

After we performed the PSM to adjust for the patients’ demographic and general characteristics, we did not observe significant differences between the characteristics of the HA and NHA groups (Table 3). However, after the PSM, we found that there were no significant differences in the two groups’ values for SBP and the DBP (Table 4). The NHA still exhibited a lower GCS, as well as higher values for HR and EMR-ISS. The NHA group also exhibited greater mortality and more prolonged hospitalizations.

**Table 3:** Baseline characteristics and demographics feature of patients after PSM.

Characteristics	Total		NHA		HA		*P*-value
	N	%	N	%	N	%	
Total	2,032	100.0	1,016	100.0	1,016	100.0	
Gender							0.934
Male	1,875	92.3	938	92.3	937	92.2	
Female	157	7.7	78	7.7	79	7.8	
Age (Years)							0.658
Mean ± SD	42.1±21.8		42.3±23.3		41.9±20.1		
Education	370		201		169		0.301
never go to school	23	6.2	11	5.5	12	7.1	
elementary school graduation	64	17.3	41	20.4	23	13.7	
middle school graduation	84	22.7	52	25.9	32	18.9	
high school graduation	111	30.0	51	25.4	60	35.5	
technical college graduation	4	1.1	2	1.0	2	1.2	
university graduation	25	6.8	12	6.0	13	7.7	
more than university	2	0.5	1	0.5	1	0.6	
unknown	57	15.4	31	15.4	26	15.4	
Insurance							0.121
NHI	675	33.2	357	35.1	318	31.3	
MAP	51	2.5	28	2.8	23	2.3	
Etc	1,306	64.3	631	62.1	675	66.4	
Unknown	0	0.0	0	0.0	0	0.0	
Method to come to hospital							0.067
Ground ambulance	1,528	75.2	780	76.8	748	73.6	
Air ambulance	6	0.3	4	0.4	2	0.2	
Etc	498	24.5	232	22.8	266	26.2	
Unknown	0	0.0	0	0.0	0	0.0	
Alcohol ingestion							0.209
No	1,836	90.4	919	90.5	917	90.3	
Yes—Only driver	193	9.5	94	9.3	99	9.7	
Yes—All rider	3	0.1	3	0.3	0	0.0	
Role of patient							0.571
Driver	1,951	96.0	978	96.3	973	95.8	
Passenger	81	4.0	38	3.7	43	4.2	
Time of accident (24hr)							0.937
06–12	424	20.9	216	21.3	208	20.5	
12–18	679	33.4	334	32.9	345	34.0	
18–24	610	30.0	304	29.9	306	30.1	
00–06	319	15.7	162	15.9	157	15.5	

*Notes*: NHA: Not worn a Helmet; HA: Worn a Helmet; NHI: National Health Insurance; MAP: Medical Aid Program

**Table 4:** Patient status and results of treatment after PSM, Mean ± SD.

Results	Total		NHA		HA		*P*-value
	N	%	N	%	N	%	
SBP (mmHg)	1,661		802		859		
Mean ± SD	133.9±26.8		133.4±28.7		134.4±24.9		0.450
DBP (mmHg)	1,661		802		859		
Mean ± SD	79.6±16.1		79.0±16.7		80.1±15.6		0.166
Heart rate	1,661		802		859		
Mean ± SD	82.9±17.2		84.0±18.7		81.9±15.6		0.014
Respiratory rate	1,659		801		858		
Mean ± SD	19.1±3.9		19.0±4.4		19.1±3.4		0.989
Glasgow coma scale	1,307		647		660		
Mean ± SD	14.2±2.6		13.9±2.9		14.4±2.3		0.005
EMR-ISS	1,451		718		733		
Mean ± SD	18.3±15.3		20.2±17.1		16.5±13.0		<0.001
Results of ER treatment	2,032		1,016		1,016		<0.001
Discharge	1,079	53.1	482	47.4	597	58.8	
Transfer	216	10.6	123	12.1	93	9.2	
Admission	699	34.4	383	37.7	316	31.1	
Death	37	1.8	27	2.7	10	1.0	
Etc	1	0.1	1	0.1	0	0.0	
Results of admission	699		383		316		0.046
Discharge	487	69.7	256	66.8	231	73.1	
Transfer to other hospital	140	20.0	74	19.3	66	20.9	
Death	56	8.0	42	11.0	14	4.4	
Etc	16	2.3	11	2.9	5	1.6	
Admission date (days)	691		635		56		
Median (IQR)	13.0 (7.0-,23.0)		14.0 (8.0-,24.0)		7.0 (3.0-,13.5)		<0.001
Death in hospital	2,032						<0.001
No	1,939	95.4	947	93.2	992	97.6	
Yes	93	4.6	69	6.8	24	2.4	

*Notes*: SBP: Systolic Blood Pressure; DBP: Diastolic Blood Pressure; EMR-ISS: Electrical Medical Records adjusted Injury Severity Score; ER: Emergency Room; SD: Standard Deviation; IQR: Interquartile Range

We did multivariate logistic regression analyses using the original and PSM datasets (Table 5). Sex, age, Insurance, Method to come to the hospital, role of patients, alcohol ingestion, and time of accident were all used in the model. No interactions were found to have significant effects and were excluded from the final model. The final model was found to have adequate calibration (P = 0.99 in original dataset, P = 1.00 in PSM dataset). In both analyses, the HA exhibited a lower risk of mortality, compared to the NHA group (original dataset, odds ratio [OR]: 0.37, 95% confidence interval [CI]: 0.23–0.59; PSM dataset, OR: 0.34, 95% CI: 0.21–0.56). A greater risk of mortality was also associated with older age and patient transfer using an air ambulance. Other forms of patient transportation (e.g., a normal vehicle or walking) were associated with a lower risk of mortality. The original dataset revealed associations of a lower risk of mortality with other insurance types (e.g., private or vehicle insurance) and alcohol consumption. However, these associations were not observed when we analyzed the PSM dataset.

**Table 5:** Logistic regression analysis on mortality in original and PSM dataset.

	Original dataset (N = 2,536)			PSM dataset (N = 2,032)		
	OR	95%CI	*P* value	OR	95%CI	*P* value
Helmet apply (ref = non)	0.37	0.23–0.59	<0.001	0.34	0.21–0.56	<0.001
Sex (ref = male)	1.01	0.54–1.87	0.984	0.80	0.36–1.78	0.589
Age	1.03	1.02–1.04	<0.001	1.03	1.01–1.04	<0.001
Insurance (ref = NHI)						
MAP	0.81	0.19–3.56	0.786	1.02	0.23–4.58	0.977
Etc	1.66	1.06–2.62	0.028	1.55	0.93–2.56	0.090
Method to come to the hospital (ref = Ground ambulance)						
Air ambulance	7.96	1.75–36.2	0.007	15.32	2.84–82.5	0.001
Etc	0.21	0.09–0.48	<0.001	0.11	0.04–0.37	<0.001
Role of patient (ref = driver)	1.06	0.53–2.15	0.865	1.38	0.45–4.20	0.569
Alcohol ingestion (ref = non)	0.48	0.24–0.94	0.033	0.52	0.20–1.32	0.167
Time of accident(ref = 06–12)						
12–18	0.98	0.60–1.60	0.946	0.80	0.48–1.35	0.401
18–24	0.62	0.32–1.20	0.153	0.55	0.27–1.10	0.091
00–06	1.38	0.73–2.60	0.318	0.88	0.40–1.93	0.751

*Notes*: NHI: National Health Insurance; MAP: Medical Aid Program; ER: Emergency Room

## Discussion

The present study aimed to evaluate whether helmet wearing was associated with mortality, severity, and hospitalization duration among patients who had only experienced a CCT after a motorcycle accident. When we compared the HA and NHA cases, we observed that HA status was associated with a lower risk of mortality after the PSM and multivariate logistic regression analyses for adjustment of the patients’ demographic and general characteristics. In addition, through the univariate analyses, we found HA status was associated with less severe injuries and shorter hospitalizations. These results agree with the findings of previous studies [4] [8–12]. Jou et al. reported that mortality was more strongly associated with NHA status, compared to HA status (OR: 3.81, 95% CI: 3.4–4.27) [11]. Keng reported that helmets can reduce the probability of death by 71% [4]. These studies showed similar conclusions as our study. However, those data was based on the national police agency database that contains only deaths within 24 hours. The data included in our study are based on medical records, and mortality rates are based on death at the time of discharge from the hospital, allowing for a more accurate mortality analysis. Furthermore, these studies included study population independent of the injured body part by motorcycle accident, not only had CCT. Dutra et al. also analyzed head and neck injury severity using GCS, and found that NHA patients had significantly lower GCS values, compared to HA patients (GCS of <13: 13.4% vs. 50.0%, *P* < 0.001) [12]. Similar to ours, this study only covered CCT, but no mortality analysis has been conducted, and the number of subjects was limited to 188.

Some previous studies have used time-series designs to compare mortality rates before and after legislation that required motorcyclists to wear helmets [13–18]. For example, Tsai and Hemenway reported a 14% decrease in overall motorcycle accident-related mortality and especially a 22% decrease in CCT-related mortality, after Taiwan passed a helmet wearing law in June 1997 [16]. In addition, Servadei et al. reported that the prevalence of helmet wearing increased from 20% to 96% and the incidence of CCT decreased by 31.4% after Italy passed a law in May 2003 requiring motorcyclists to wear helmets [18]. On the other hand, Banas and Knudson compared each state of United States of America that did and did not have helmet wearing laws in 2001 and found that there was a significant difference in univariate analysis between mortality and a helmet-wearing law, although the association was not significant in the multivariate analysis [19]. However, that study only included the amount of alcohol consumption and age as covariates in the multivariate analysis, which is not sufficient for the adjustment of the difference in patients’ demographic and general characteristics.

The present study has two strengths, compared to previous studies. First, we included patients who had only craniocerebral trauma. Helmet wearing can minimize damage by protecting an individual’s head during a motorcycle accident. In contrast, most previous studies included all patients regardless of their injured body parts, and these studies may have included patients who died because of traumatic injury of other body parts, which is a limitation inaccurate analysis about the effect of wearing a helmet in motorcycle accident. Second, we used PSM to adjust for the patients’ demographic and clinical characteristics. The un-matched univariate analyses had significant differences in various factors, including age, alcohol consumption, and time of the accident. Previous studies have detected these associations, 2011 study by Yu et al. revealed differences in age, education level, speed at the accident, and alcohol consumption when they compared the HA and NHA groups [20]. Although they adjusted for those differences using multiple logistic regressions, our study had designed with more powerful analysis by using PSM for adjustment of variables which can influence the result except wearing helmets.

South Korea has a law that requires motorcyclists to wear a helmet or other protective equipment. However, the 2014 Road Safe Annual Report indicated that only 78% of South Korean motorcyclists wore helmets, and this result was much lower than the rates of approximately 100% in European countries [21]. Therefore, both legislation and practical interventions are needed to increase the prevalence of helmet wearing in South Korea. Especially, teenagers have a very low rate of helmet wearing, and education efforts should target this age group.

The present study had several limitations. First, the data were retrospectively collected from various university hospitals and this approach is prone to selection bias, as we could not have enrolled enough data from vulnerable rural areas. Second, we could not analyze some variables that are relevant when evaluating motorcycle accidents. For example, we did not have access to data regarding the type of helmet or the motorcycle’s speed at the accident. Third, we only evaluated mortality data from hospitals that are included in the EDIIS database, and we could not analyze mortality among patients who were transferred after acute management. Finally, we only evaluated mortality and severity as clinical outcomes, and we are unable to comment on the survivors’ quality of life.

## Conclusion

Wearing a helmet may help reduce the risk of mortality from a CCT after a motorcycle accident. Furthermore, wearing a helmet was associated with reduced severity and shorter hospitalization.

## Supporting information

S1 Table Included ICD-10 diagnostic code of CCT. (DOCX)
