# Supplementary material for: Can helmet decrease mortality of craniocerebral trauma patients in a motorcycle accident?: A propensity score matching
Source: PLoS One. 2020 Jan 13;15(1):e0227691. doi: 10.1371/journal.pone.0227691 (PMC6957151; doi:10.1371/journal.pone.0227691)
Supplement: S1 Table — (DOCX) [file pone.0227691.s001.docx]

**Table. Included ICD-10 diagnostic code of CCT**

| **ICD-10 Code** | **Diagnosis** |
| --- | --- |
| S01.0 | Open wound of scalp |
| S01.1 | Open wound of eyelid and periorbital are |
| S01.2 | Open wound of nose |
| S01.3 | Open wound of ear |
| S01.4 | Open wound of cheek and temporomandibular area |
| S01.5 | Open wound of lip and oral cavity |
| S01.7 | Multiple open wound of head |
| S01.8 | Open wound of other parts of head |
| S01.9 | Open wound of head, part unspecified |
|  |  |
| S02.0 | Fracture of vault of skull |
| S02.1 | Fracture of base of skull |
| S02.3 | Fracture of orbital floor |
| S02.7 | Multiple fractures involving skull and facial bones |
| S02.8 | Fracture of other skull and facial bones |
| S02.9 | Fracture of skull and facial bones, part unspecified |
|  |  |
| S04.0 | Injury of optic nerves and pathways |
|  |  |
| S06.0 | Concussion |
| S06.1 | Traumatic cerebral oedema |
| S06.3 | Focal brain injury |
| S06.4 | Epidural hemorrhage (Traumatic extradural hemorrhage) |
| S06.5 | Traumatic subdural hemorrhage |
| S06.6 | Traumatic subarachnoid hemorrhage |
| S06.7 | Intracranial injury with prolonged coma |
| S06.8 | Other intracranial injuries |
| S06.9 | Intracranial injury, unspecified |
| S07.0 | Crushing injury of face |
| S07.1 | Crushing injury of skull |
| S07.8 | Crushing injury of other parts of head |
| S07.9 | Crushing injury of head, part unspecified |
|  |  |
| S09.7 | Multiple injuries of head |
| S09.8 | Other specified injuries of head |
| S09.9 | Unspecified injury of head |
|  |  |
| T01.0 | Open wounds involving head with neck |
| T02.0 | Fractures involving head with neck |
| T04.0 | Crushing injuries involving head with neck |
| T06.0 | Injuries of brain and cranial nerves with injuries of nerves and spinal cord at neck level |
